# Supplementary material for: Risk and adverse outcomes of fractures in patients with liver cirrhosis: two nationwide retrospective cohort studies
Source: BMJ Open. 2017 Oct 8;7(10):e017342. doi: 10.1136/bmjopen-2017-017342 (PMC5640047; doi:10.1136/bmjopen-2017-017342)
Supplement: Author's manuscript [file bmjopen-2017-017342.draft_revisions.pdf]

BMJ Open

BMJ Open is committed to open peer review. As part of this commitment we make the peer review history of every article we publish publicly available.

When an article is published we post the peer reviewers' comments and the authors' responses online. We also post the versions of the paper that were used during peer review. These are the versions that the peer review comments apply to.

The versions of the paper that follow are the versions that were submitted during the peer review process. They are not the versions of record or the final published versions. They should not be cited or distributed as the published version of this manuscript.

BMJ Open is an open access journal and the full, final, typeset and author-corrected version of record of the manuscript is available on our site with no access controls, subscription charges or pay-per-view fees (http://bmjopen.bmj.com).

If you have any questions on BMJ Open's open peer review process please email editorial.bmjopen@bmj.com

BMJ Open

Risk and adverse outcomes of fractures in patients with liver cirrhosis: two nationwide retrospective cohort studies

Journal:	*BMJ Open*
Manuscript ID	bmjopen-2017-017342
Article Type:	Research
Date Submitted by the Author:	20-Apr-2017
Complete List of Authors:	Chen, Ta-Liang; Taipei Medical University Hospital, Department of Anesthesiology<br>Lin, Chao-Shun; Taipei Medical University Hospital, Department of Anesthesiology<br>Shih, Chun-Chuan; I-Shou University, School of Chinese Medicine for Post-Baccalaureate<br>Huang, Yu-Feng; Mackay Memorial Hospital Taitung Branch Hospital, Department of Anesthesiology<br>Yeh, Chun-Chieh; University of Illinois, Department of Surgery<br>Wu, Chih-Hsing; National Cheng Kung University Hospital, Family Medicine<br>Liao, Chien-Chang; Taipei Medical University Hospital, Department of Anesthesiology; Taipei Medical University, Department of Anesthesiology, School of Medicine
**Primary Subject Heading**:	Epidemiology
Secondary Subject Heading:	Gastroenterology and hepatology, Rehabilitation medicine
Keywords:	liver cirrhosis, fracture, risk, outcome

SCHOLARONE™
Manuscripts

Risk and adverse outcomes of fractures in patients with liver cirrhosis: two nationwide retrospective cohort studies

Ta-Liang Chen,[1,2,3] Chao-Shun Lin,[1,2,3] Chun-Chuan Shih,[4] Yu-Feng Huang,[5] Chun-Chieh Yeh,[6,7] Chih-Hsing Wu,[8] Chien-Chang Liao[1,2,3,9,10]

[1]Department of Anesthesiology, Taipei Medical University Hospital, Taipei, Taiwan

[2]Anesthesiology and Health Policy Research Center, Taipei Medical University Hospital, Taipei, Taiwan

[3]Department of Anesthesiology, School of Medicine, College of Medicine, Taipei Medical University, Taipei, Taiwan

[4]The School of Chinese Medicine for Post-Baccalaureate, I-Shou University, Kaohsiung, Taiwan

[5]Department of Anesthesiology, Taitung Mackay Memorial Hospital, Taitung, Taiwan

[6]Department of Surgery, China Medical University Hospital, Taichung, Taiwan

[7]Department of Surgery, University of Illinois, Chicago, United States of America

[8]Department of Family Medicine, National Cheng Kung University Hospital, Tainan, Taiwan

[9]School of Chinese Medicine, College of Chinese Medicine, China Medical University, Taichung, Taiwan

[10]Department of Anesthesiology, Shuan Ho Hospital, Taipei Medical University, New Taipei City, Taiwan

For peer review only

For peer review only - http://bmjopen.bmj.com/site/about/guidelines.xhtml

Correspondence:

Chien-Chang Liao, PhD, MPH

Associate Professor

Department of Anesthesiology, Taipei Medical University Hospital

252 Wuxing St., Taipei 11031, Taiwan

Tel: 886-2-2737-2181, ext. 8310

Fax: 886-2-2736-7344

E-mail: jacky48863027@yahoo.com.tw; ccliao@tmu.edu.tw

Abstract: 230 words; Text: 2243 words; References: 39

Running head: Liver cirrhosis and fracture

Abbreviations: CI=confidence interval; HR=hazard ratio; ICD-9-CM=International Classification of Diseases, Ninth Revision, Clinical Modification; LC=liver cirrhosis; OR=odds ratio

Key Words: liver cirrhosis; fracture; risk; outcomes

For peer review only

Abstract

Objective: The aim of this study is to evaluate fracture risk and post-fracture outcomes in patients with liver cirrhosis (LC).

Design: Retrospective cohort study and nested cohort study.

Setting: This study was based on Taiwan's National Health Insurance Research Database that included information on: (1) 3941patients with aged 20 years and older newly diagnosed with LC between 2000 and 2003; (2) 688,560 hospitalized fracture patients aged 20 years and older between 2006 and 2013.

Primary and secondary outcome measures: Followed-up events of fracture from 2000 until 2008 were noted from medical claims to evaluate adjusted hazard ratios (HRs) and 95% confidence intervals (CIs) of fracture associated with LC. Adjusted odds ratios (ORs) and 95% CIs of adverse events after fracture were compared among patients with and without LC

Results: The incidences of fracture for people with and without LC were 28.0 and 16.9 per 1000 person-years, respectively. Compared with control, the adjusted HR of fracture was 1.71 (95% CI 1.55-1.87) for LC patients. Previous LC was associated with risks of septicemia (OR 1.87, 95 % CI 1.68-2.0), acute renal failure (OR 1.77, 95% CI 1.43-2.18), and mortality (OR 1.71, 95 % CI 1.45-2.01) after fracture.

Conclusion: LC was associated with higher risk of fracture; LC patients in particular had more complications and mortality after fracture. Fracture prevention and attention to

For peer review only - http://bmjopen.bmj.com/site/about/guidelines.xhtml

post-fracture adverse events are needed for these susceptible populations.

For peer review only

For peer review only - http://bmjopen.bmj.com/site/about/guidelines.xhtml

Strengths of this study

(1) Our study is an longitudinal retrospective cohort with large sample size.

(2) We used frequency matching and multivariate Cox proportional hazard models to control the confounding bias.

(3) This is the first study with comprehensive assessment of the impact of liver cirrhosis on the fracture risk and outcomes.

Limitations of this study

(1) Our data lack of clinical risk scores, lesion characteristics, biochemical measures, and lifestyles of fracture patients and cirrhosis patients.

(2) The detailed lifestyle included smoking, alcohol drinking, and physical activity were not available.

For peer review only

INTRODUCTION

Liver cirrhosis (LC) is the fourth-most-common cause of death in Europe, and causes more than one million deaths every year worldwide.[1,2] Prevalence of LC increased 68% during the 1992-2001 decade in the UK; in the US, it remains a pandemic chronic disease with more than 600,000 patients in 1999-2010 whose economic burden was shown to have doubled during 1998 and 2003.[3-5] Although the epidemiology, pathogenesis, prevention, and treatment of LC have been studied,[6] complications of liver cirrhosis are not well understood.

Fracture causes disability and mortality, and places economic burdens on societies.[7,8] Since many studies found patients with LC had increased risk of osteoporosis,[9-11] fall and fracture were considered complications for patients with LC.[12-20] However, these studies did not compare the risk of fracture or falls between people with and without LC. Small sample size,[12,14-18] inadequate adjustment for confounding factors,[12-18] case-control study design,[12,14,16-19] focus on specific population,[12] lack of control group,[14,16,18,19] and of subgroup analysis [14-17,19,20] limited previous investigations. Information also was lacking on whether LC is associated with post-fracture adverse outcomes.

With the use of reimbursement claims from Taiwan's National Health Insurance program, we conducted two nationwide cohort studies. The retrospective cohort study seeks to validate

For peer review only - http://bmjopen.bmj.com/site/about/guidelines.xhtml

the risk of fracture in patients with LC. Whether LC was associated with adverse outcomes after fracture was reported in the nested cohort study.

For peer review only

For peer review only - http://bmjopen.bmj.com/site/about/guidelines.xhtml

METHODS

Source of data

This study used the claims data of Taiwan's National Health Insurance Program, which was implemented in March 1995 and covers 99% of 23 million people nationwide. The National Health Research Institutes (NHRI) established the National Health Insurance Research Database to record all beneficiaries' information about inpatient and outpatient medical services. This includes basic patient demographics, physician's primary and secondary diagnoses, treatment procedures, prescribed medications, and medical expenditures. All contracted medical clinics, hospitals and medical centers are required to submit computerized claim documents for medical expenses. The validity of this database has been favorably evaluated, and research articles based on it have been accepted in scientific journals worldwide.[21-23]

Ethical approval

Insurance reimbursement claims used in this study were decoded with patient identifications scrambled for further research access. This study was conducted inaccordance with the Helsinki Declaration. Although NHRI regulations do not require informed consent because patient identification has been decoded for privacy, this study also was approved by Taiwan's National Health Research Institutes (NHIRD-103-121) and Taipei Medical University's Joint Institutional Review Board (TMU-JIRB-201404070).

For peer review only - http://bmjopen.bmj.com/site/about/guidelines.xhtml

Study design

From the representative sample of 1,000,000 insurance enrollees, we required at least two visits for medical care with the physician's primary diagnosis of cirrhosis of the liver to identify a cohort of 3941 newly diagnosed adults aged ≥ 20 years in 2000-2003. The frequency-matching procedure (by age and sex) was used to select the cohort, who had no previous medical records of LC. Both LC and non-LC cohorts had no history of fracture between the index date (date of second LC diagnosis) and January 1, 1996 (the starting date of the Taiwan's National Health Insurance Program). The outcome of this retrospective cohort study is incident event of fracture that was identified during the follow-up period from the index date until the end of 2008 for LC and non-LC cohorts. The purpose of this study is to compare the risk of fracture between people with and without LC.

In the fracture nested cohort study including 688,560 hospitalized fracture patients in 2004-2013, we identified 7854 with history of LC within pre-fracture 24 months. Thirty-day mortality, septicemia, and acute renal failure after fracture were considered as post-fracture outcomes and compared between fracture patients with and without LC.

Measurements and definitions

Low-income status was determined by the National Health Insurance Bureau, which validates those qualified for waived health-care co-payment. Following previous suggestions,[21,22] fracture-associated medications were also analyzed; these included

For peer review only - http://bmjopen.bmj.com/site/about/guidelines.xhtml

anxiolytics, antiepileptics, antipsychotics, antidepressants, and oral steroids. We used the *International Classification of Diseases, Ninth Revision, Clinical Modification* (ICD-9-CM) and administration codes of Taiwan's National Health Insurance to examine the physician's diagnoses and medical services. We identified patient medical conditions and complications such as mental disorders (ICD-9-CM 290-319), hypertension (ICD-9-CM 410-405), chronic obstructive pulmonary disease (ICD-9-CM 490-496), diabetes (ICD-9-CM 250), ischemic heart disease (ICD-9-CM 410-414), stroke (ICD-9-CM 430-438), hyperlipidemia (ICD-9-CM 272.0, 272.1, and 272.2), heart failure (ICD-9-CM 428), renal dialysis (D8, D9), Parkinson's disease (ICD-9-CM 332), liver cirrhosis (571.2, 571.5, and 571.6), fracture (ICD-9-CM 800-829), sepsis (ICD-9-CM 038 and 998.5), and acute renal failure (ICD-9-CM 548). Thirty-day in-hospital mortality is the main outcome in the nested fracture cohort study.

Statistical analysis

In the retrospective cohort study, the categorical data for cohorts with and without LC were analyzed by chi-square tests. The adjusted hazard ratios (HRs) and confidence intervals (CIs) of fracture risk associated with liver cirrhosis were calculated in the multiple Cox proportional hazard models, as were associations between LC and fracture risk in males, females, and every age group. We used chi-square tests to examine other sociodemographic factors and medical conditions in the nested fracture study. The multiple logistic regressions were used to calculate adjusted odds ratios (ORs) and 95% CIs of post-fracture mortality,

For peer review only - http://bmjopen.bmj.com/site/about/guidelines.xhtml

sepsis, and acute renal failure associated with history of LC.

For peer review only

For peer review only - http://bmjopen.bmj.com/site/about/guidelines.xhtml

RESULTS

Because we used frequency-matching procedure (Table 1), there was no significant difference in age and sex between cohorts with and without LC. The LC cohort had more medical conditions than the non-LC cohort; these included mental disorders ($p<0.0001$), hypertension ($p=0.0046$), chronic obstructive pulmonary disease ($p<0.0001$), diabetes ($p<0.0001$), ischemic heart disease ($p<0.0001$), stroke ($p<0.0001$), hyperlipidemia ($p=0.0003$), heart failure ($p<0.0001$), renal dialysis ($p<0.0001$), and Parkinson's disease ($p<0.0001$). The use of anxiolytics ($p<0.0001$), antiepileptics ($p<0.0001$), antipsychotics ($p<0.0001$), and antidepressants ($p<0.0001$) was also higher in patients with LC than in people without LC.

During the 5-8 years of follow-up (Table 2), the incidences of fracture for cohorts with and without LC were 29.1 and 17.2 per 1000 person-years, respectively. The increased risk of fracture was found in LC cohort after adjustment (HR 1.83, 95% CI 1.67-2.01). We also found significant associations between fracture risk and LC in males (HR 2.04, 95% CI 1.81-2.31), females (HR 1.53, 95% CI 1.32-1.78), and people aged 20-39 (HR 2.12, 95% CI 1.59-2.81), 40-49 (HR 2.04, 95% CI 1.63-2.54), 50-59 (HR 1.67, 95% CI 1.34-2.07), 60-69 (HR 1.69, 95% CI 1.38-2.06), and ≥70 years (HR 1.70, 95% CI 1.41-2.03).

Among 688,560 hospitalized fracture patients, patients with LC had lower proportions of young adults ($p<0.0001$) but higher proportions of males ($p<0.0001$) and low-income status ($p<0.0001$). More history of mental disorders, diabetes, chronic obstructive pulmonary

For peer review only - http://bmjopen.bmj.com/site/about/guidelines.xhtml

disease, ischemic heart disease, stroke, congestive heart failure, Parkinson's disease, and renal dialysis were found in patients with LC than in control (p<0.05 for all). Fracture patients with previous LC had higher proportions of neck or trunk fracture (p<0.0001) and lower limb fracture (p<0.0001).

Higher risks for post-fracture sepsis (OR 1.76, 95% CI 1.59-1.95), acute renal failure (OR 1.67, 95% CI 1.37-2.04), and 30-day in-hospital mortality (OR 1.64, 95% CI 1.40-1.93) were associated with previous LC (Table 4). Fracture patients with LC had higher mean length of hospital stay (9.6±10.5 vs. 8.5±13.8 days, p<0.0001) and medical expenditure (2500±2743 vs. 2212±2550 USD, p<0.0001) than patients without LC.

Previous LC was associated with higher risks of adverse events after fracture (Table 5) in females (OR 2.01, 95% CI 1.75-2.32), males (OR 1.62, 95% CI 1.46-1.80), and patients aged 30-39 (OR 1.82, 95% CI 1.34-2.49), 40-49 (OR 2.25, 95% CI 1.83-2.75), 50-59 (OR 1.89, 95% CI 1.53-2.34), 60-69 (OR 1.99, 95% CI 1.59-2.48), and ≥70 years (OR 1.42, 95% CI 1.25-1.62). Associations between post-fracture adverse events and LC were significant in patients with fracture receiving surgery (OR 2.17, 95% CI 1.92-2.45), traumatic brain injury (OR 1.60, 95% CI 1.35-1.90), or fracture of upper limb (OR 1.77, 95% CI 1.44-2.17), lower limb (OR 1.80, 95% CI 1.61-2.02), and neck or trunk (OR 1.42, 95% CI 1.16-1.73).

Among fracture patients with LC (Table 6), alcohol dependence syndrome (OR 1.96, 95% CI 1.57-2.44), jaundice (OR 3.17, 95% CI 2.44-4.13), ascites (OR 2.52, 95% CI 2.10-3.01),

For peer review only - http://bmjopen.bmj.com/site/about/guidelines.xhtml

gastrointestinal hemorrhage (OR 2.10, 95% CI 1.80-2.46), and hepatic coma (OR 2.80, 95% CI 2.30-3.41) were significant determinants for post-fracture adverse events. A biological gradient relationship was found between number of cirrhotic indicators and post-fracture adverse events (OR 2.87, 95% CI 1.60-5.15).

For peer review only

For peer review only - http://bmjopen.bmj.com/site/about/guidelines.xhtml

DISCUSSION

In this retrospective cohort study among adult patients, we observed a significant association between LC and fracture, with an 83% increased risk of fracture in patients with LC during the 5-8 years of follow-up. We also observed that patients with LC had significantly higher post-fracture complications and mortality in the nested cohort study. Clinical indicators of the severity of LC, such as alcohol dependence syndrome, jaundice, ascites, gastrointestinal hemorrhage, and hepatic coma were all associated with more post-fracture adverse events.

In general, the prevalence of LC was higher in males than in females.[4,24] Older age and low socioeconomic status were factors associated with higher risk of LC.[3,23,25] Older age, males, and low income were also risk factors for fracture, and were adjusted by using the multivariate Cox proportional models to control these potential confounding effects for the analysis of association between LC and fracture risk.[26] Furthermore, we found that the association between LC and fracture risk remains significant in every age group and both sexes. The significant impact of LC on post-fracture adverse events was noted in males, females, various age, and people with various types of fracture. This phenomenon revealed the possible causal inference that LC was associated with fracture risk and post-fracture adverse events from the viewpoint of epidemiology. These findings were crucial because several previous studies were limited by focusing on specific population by failing to

For peer review only

investigate the association in subgroup analysis.[14-17,19,20]

Mental disorders, hypertension, chronic obstructive pulmonary disease, diabetes, ischemic heart disease, stroke, hyperlipidemia, congestive heart failure, renal dialysis and Parkinson's disease were considered as co-existing medical conditions that also were fracture risk factors.[21,22,27-29,30-32] Confounding bias may occurred in previous studies that lacked multivariate adjustment for these fracture-related and/or cirrhosis-related medical conditions. These were adjusted using multiple Cox proportional hazard and multiple logistic regression models to investigate the risks and outcomes of fracture in patients with LC.[12-18]

Unlike previous investigations, we studied the impact of LC on post-fracture outcomes such as septicemia, acute renal failure, and mortality.[12-20] Fracture patients with history of LC had longer hospital stay and medical expenditure than non-LC people in the nested cohort study. Patients with LC had circulatory dysfunction and poor immune systems that compromise systemic inflammatory response and make them prone to renal failure and septicemia,[23,33] particularly those patients with cirrhotic indicators such as alcohol dependence syndrome, jaundice, ascites, gastrointestinal hemorrhage, and hepatic coma. Therefore, higher mortality and consumption of medical resources might be encountered in the LC population during fracture admissions.

Associations between LC and fracture risk suggest several possible explanations. First, many studies found LC patients had increased risk of osteoporosis,[9-11] a condition that is

For peer review only - http://bmjopen.bmj.com/site/about/guidelines.xhtml

an important determinant for fracture.[8] Fracture due to bone loss and the pathogenesis of osteoporosis among patients with LC is complex and multifactorial, and the exact mechanism remains uncertain. A previous study showed cirrhotic patients with osteoporosis had lower levels of insulin-like growth factor-1 than cirrhotic patients without osteoporosis.[34] Insulin-like growth factor-1 plays a major role in bone remodeling and maintenance of bone mass, and was found to be reduced in advanced cirrhosis.[35] Cirrhotic patients' hyperbilirubinemia also has been shown to impair osteoblast proliferation, resulting in decreased bone formation and possibly accounting for the increased risks of fracture.[36] Second, corticosteroids are frequently used in patients with autoimmune hepatitis and other inflammatory disorders. Even budesonide, a corticosteroid with minimal systemic availability, might lead to accelerated bone loss in cirrhotic patients and postmenopausal women.[37] We postulated that medications used in the treatment of LC could also have an adverse effect on bone and calcium mobilization and subsequent osteoporosis. Third, hepatic coma, poor cognitive function, and psychiatric illness may play roles in the association between LC and risk of fracture.[25,38] Though hepatic encephalopathy does not commonly occur in patients with LC, its contribution to falls should not be ignored.[39]

Some study limitations need to be addressed when interpreting the present results. First, this study used retrospective reimbursement claims, which lack data on severity of LC, lifestyle factors, personal characteristics, and biochemical data. Second, since the patients

For peer review only

were selected based on diagnoses from hospital inpatient care registers, patients with minor LC but no symptoms might not seek medical services, leading to underestimation of fracture risk in LC patients because some minor LC patients may have been in the non-LC group. Third, because our results are based on the data from Taiwan's National Health Insurance, findings of this study could not be directly generalized to other populations.

Conclusion

Our two cohort studies provide population-based evidence that LC is an important risk factor for fracture. We also noted that fracture patients with various clinical indicators of LC severity face increased risks of post-fracture adverse events. We demonstrated risk factor analysis and a variety of clinical suggestions, including prevention, risk assessment and outcome-related information to fracture patients with LC. Strategies to prevent fracture and meticulous care to reduce post-fracture adverse events should be routinely considered for this population.

For peer review only

For peer review only - http://bmjopen.bmj.com/site/about/guidelines.xhtml

REFERENCES

1. Lozano R, Naghavi M, Foreman K, Lim S, Shibuya K, Aboyans V, et al. Global and regional mortality from 235 causes of death for 20 age groups in 1990 and 2010: a systematic analysis for the Global Burden of Disease Study 2010. *Lancet.* 2012;380:2095-128.
2. Blachier M, Leleu H, Peck-Radosavljevic M, Valla DC, Roudot-Thoraval F. The burden of liver disease in Europe: a review of available epidemiological data. *J Hepatol.* 2013;58:593-608.
3. Scaglione S, Kliethermes S, Cao G, Shoham D, Durazo R, Luke A, et al. The epidemiology of cirrhosis in the United States: a population-based study. *J Clin Gastroenterol.* 2015;49:690-6.
4. Fleming KM, Aithal GP, Solaymani-Dodaran M, Card TR, West J. Incidence and prevalence of cirrhosis in the United Kingdom, 1992-2001: a general population-based study. *J Hepatol.* 2008;49:732-8.
5. Nguyen GC, Segev DL, Thuluvath PJ. Nationwide increase in hospitalizations and hepatitis C among inpatients with cirrhosis and sequelae of portal hypertension. *Clin Gastroenterol Hepatol.* 2007;5:1092-9.
6. Tsochatzis EA, Bosch J, Burroughs AK. Liver cirrhosis. *Lancet.* 2014;383:1749-61.
7. Bliuc D, Nguyen ND, Milch VE, Nguyen TV, Eisman JA, Center JR. Mortality risk

For peer review only

For peer review only - http://bmjopen.bmj.com/site/about/guidelines.xhtml

associated with low-trauma osteoporotic fracture and subsequent fracture in men and women. *JAMA.* 2009;301:513-21.

8. Burge R, Dawson-Hughes B, Solomon DH, Wong JB, King A, Tosteson A. Incidence and economic burden of osteoporosis-related fractures in the United States, 2005-2025. *J Bone Miner Res.* 2007;22:465-75.

9. Lupoli R, Di Minno A, Spadarella G, Ambrosino P, Panico A, Tarantino L, et al. The risk of osteoporosis in patients with liver cirrhosis: a meta-analysis of literature studies. *Clin Endocrinol.* 2016;84:30-8.

10. Guañabens N, Parés A, Ros I, Caballería L, Pons F, Vidal S, et al. Severity of cholestasis and advanced histological stage but not menopausal status are the major risk factors for osteoporosis in primary biliary cirrhosis. *J Hepatol.* 2005;42:573-7.

11. Mounach A, Ouzzif Z, Wariaghli G, Achemlal L, Benbaghdadi I, Aouragh A, et al. Primary biliary cirrhosis and osteoporosis: a case-control study. *J Bone Miner Metab.* 2008;26:379-84.

12. Boulton-Jones JR, Fenn RM, West J, Logan RF, Ryder SD. Fracture risk of women with primary biliary cirrhosis: no increase compared with general population controls. *Aliment Pharmacol Ther.* 2004;20:551-7.

13. Bang UC, Benfield T, Bendtsen F, Hyldstrup L, Beck Jensen JE. The risk of fractures among patients with cirrhosis or chronic pancreatitis. *Clin Gastroenterol Hepatol.*

For peer review only

For peer review only - http://bmjopen.bmj.com/site/about/guidelines.xhtml

2014;12:320-6.

14. Román E1, Córdoba J, Torrens M, Guarner C, Soriano G. Falls and cognitive dysfunction impair health-related quality of life in patients with cirrhosis. *Eur J Gastroenterol Hepatol.* 2013;25:77-84.

15. Solaymani-Dodaran M, Card TR, Aithal GP, West J. Fracture risk in people with primary biliary cirrhosis: a population-based cohort study. *Gastroenterology.* 2006;131:1752-7.

16. Guañabens N, Cerdá D, Monegal A, Pons F, Caballería L, Peris P, et al. Low bone mass and severity of cholestasis affect fracture risk in patients with primary biliary cirrhosis. *Gastroenterology.* 2010;138:2348-56.

17. Frith J, Kerr S, Robinson L, Elliott C, Ghazala C, Wilton K, et al. Primary biliary cirrhosis is associated with falls and significant fall related injury. *QJM.* 2010;103:153-61.

18. Soriano G, Román E, Córdoba J, Torrens M, Poca M, Torras X, et al. Cognitive dysfunction in cirrhosis is associated with falls: a prospective study. *Hepatology.* 2012;55:1922-30.

19. Tapper EB, Risech-Neyman Y, Sengupta N. Psychoactive medications increase the risk of falls and fall-related injuries in hospitalized patients with cirrhosis. *Clin Gastroenterol Hepatol.* 2015;13:1670-5.

20. Tsai CF, Liu CJ, Chen TJ, Chu CJ, Lin HC, Lee FY, et al. Increased incidence of orthopedic fractures in cirrhotic patients: a nationwide population-based study. *J Hepatol.*

For peer review only

2013;58:706-14.

21. Huang YF, Cherng YG, Hsu SP, Yeh CC, Chou YC, Wu CH, et al. Risk and adverse outcomes of fractures in patients with Parkinson's disease: two nationwide studies. *Osteoporos Int.* 2015;26:1723-32.

22. Liao CC, Lin CS, Shih CC, Yeh CC, Chang YC, Lee YW, et al. Increased risk of fracture and postfracture adverse events in patients with diabetes: two nationwide population-based retrospective cohort studies. *Diabetes Care.* 2014;37:2246-52.

23. Lin CS, Lin SY, Chang CC, Wang HH, Liao CC, Chen TL. Postoperative adverse outcomes after non-hepatic surgery in patients with liver cirrhosis. *Br J Surg.* 2013;100:1784-90.

24. Scaglione S, Kliethermes S, Cao G, Shoham D, Durazo R, Luke A, et al. The epidemiology of cirrhosis in the United States: a population-based study. *J Clin Gastroenterol.* 2015;49:690-6.

25. Bajaj JS, Riggio O, Allampati S, Prakash R, Gioia S, Onori E, et al. Cognitive dysfunction is associated with poor socioeconomic status in patients with cirrhosis: an international multicenter study. *Clin Gastroenterol Hepatol.* 2013;11:1511-6.

26. Trimpou P, Landin-Wilhelmsen K, Odén A, Rosengren A, Wilhelmsen L. Male risk factors for hip fracture-a 30-year follow-up study in 7,495 men. *Osteoporos Int.* 2010;21:409-16.

27. Jepsen P, Vilstrup H, Lash TL. Development and validation of a comorbidity scoring

For peer review only - http://bmjopen.bmj.com/site/about/guidelines.xhtml

system for patients with cirrhosis. *Gastroenterology.* 2014;146:147-56.

28. Gershwin ME, Selmi C, Worman HJ, Gold EB, Watnik M, Utts J, et al. Risk factors and comorbidities in primary biliary cirrhosis: a controlled interview-based study of 1032 patients. *Hepatology.* 2005;42:1194-202.

29. Tryc AB, Goldbecker A, Berding G, Rümke S, Afshar K, Shahrezaei GH, et al. Cirrhosis-related Parkinsonism: prevalence, mechanisms and response to treatments. *J Hepatol.* 2013;58:698-705.

30. Dennison EM, Compston JE, Flahive J, Siris ES, Gehlbach SH, Adachi JD, et al. Effect of co-morbidities on fracture risk: findings from the Global Longitudinal Study of Osteoporosis in Women (GLOW). *Bone.* 2012;50:1288-93.

31. Hippisley-Cox J, Coupland C. Derivation and validation of updated QFracture algorithm to predict risk of osteoporotic fracture in primary care in the United Kingdom: prospective open cohort study. *BMJ.* 2012;344:e3427.

32. Dennison EM, Compston JE, Flahive J, Siris ES, Gehlbach SH, Adachi JD, et al. Effect of co-morbidities on fracture risk: findings from the Global Longitudinal Study of Osteoporosis in Women (GLOW). *Bone.* 2012;50:1288-93.

33. Gines P, Schrier RW. Renal failure in cirrhosis. *N Engl J Med.* 2009;361:1279-90.

34. Gallego-Rojo FJ, Gonzalez-Calvin JL, Muñoz-Torres M, Mundi JL, Fernandez-Perez R, Rodrigo-Moreno D. Bone mineral density, serum insulin-like growth factor I, and bone

For peer review only

turnover markers in viral cirrhosis. *Hepatology.* 1998;28:695-9.

35. Leslie WD, Bernstein CN, Leboff MS. AGA technical review on osteoporosis in hepatic disorders. *Gastroenterology.* 2003;125:941-66.

36. Janes CH, Dickson ER, Okazaki R, Bonde S, McDonagh AF, Riggs BL. Role of hyperbilirubinemia in the impairment of osteoblast proliferation associated with cholestatic jaundice. *J Clin Invest.* 1995;95:2581-6.

37. Angulo P, Jorgensen RA, Keach JC, Dickson ER, Smith C, Lindor KD. Oral budesonide in the treatment of patients with primary biliary cirrhosis with a suboptimal response to ursodeoxycholic acid. *Hepatology.* 2000;31:318-23.

38. Van Os E, van den Broek WW, Mulder PG, ter Borg PC, Bruijn JA, van Buuren HR. Depression in patients with primary biliary cirrhosis and primary sclerosing cholangitis. *J Hepatol.* 2007;46:1099-103.

39. Román E, Córdoba J, Torrens M, Torras X, Villanueva C, Vargas V, et al. Minimal hepatic encephalopathy is associated with falls. *Am J Gastroenterol.* 2011;106:476-82.

For peer review only

Acknowledgments: This study is based in part on data obtained from the National Health Insurance Research Database provided by the Bureau of National Health Insurance, Ministry of Health and Welfare, and managed by the National Health Research Institutes. The interpretation and conclusions contained herein do not represent those of the Bureau of National Health Insurance, Ministry of Health and Welfare, or National Health Research Institutes.

Competing interests: None.

Financial support: This research was supported in part by Shuang Ho Hospital, Taipei Medical University (104TMU-SHH-23), Taiwan's Ministry of Science and Technology (MOST105-2629-B-038-001; MOST105-2314-B-038-025; MOST104-2314-B-038-027-MY2; NSC102-2314-B-038-021-MY3), and Taiwan's Ministry of Health and Welfare Clinical Trial and Research Center of Excellence (MOHW105-TDU-B-212-133019).

Data sharing statement: dataset available from the Taiwan's National Health Research Institutes (http://nhird.nhri.org.tw/index1.php).

Author contribution: All the authors revised and approved the contents of the submitted article. TLC and CCL created the idea of the manuscript and wrote the draft. CCL conducted statistical analysis of data. All the authors made substantial contributions to interpretation of data and carried out a critical revision of the manuscript for important intellectual content.

For peer review only

Table 1 Sociodemographics, coexisting medical conditions, and medication use in people with and without liver cirrhosis

	Non-liver cirrhosis (N=15764)		Liver cirrhosis (N=3941)		*P* value
Sex	n	(%)	N	(%)	1.0000
Female	4980	(31.6)	1245	(31.6)	
Male	10784	(68.4)	2696	(68.4)	
Age, years					1.0000
20-29	620	(3.9)	155	(3.9)	
30-39	1924	(12.2)	481	(12.2)	
40-49	3628	(23.0)	907	(23.0)	
50-59	3624	(23.0)	906	(23.0)	
60-69	3320	(21.1)	830	(21.1)	
≥70	2648	(16.8)	662	(16.8)	
Low income	362	(2.3)	211	(5.3)	<0.0001
Coexisting medical conditions					
Mental disorders	3974	(25.2)	1437	(36.5)	<0.0001
Hypertension	5248	(33.3)	1406	(35.7)	0.0046
COPD	2892	(18.4)	992	(25.2)	<0.0001
Diabetes	2242	(14.2)	944	(24.0)	<0.0001
Ischemic heart disease	2623	(16.6)	788	(20.0)	<0.0001
Stroke	941	(6.0)	338	(8.6)	<0.0001
Hyperlipidemia	1468	(9.3)	294	(7.5)	0.0003
Congestive heart failure	453	(2.9)	255	(6.5)	<0.0001
Renal dialysis	123	(0.8)	112	(2.8)	<0.0001
Parkinson's disease	255	(1.6)	105	(2.7)	<0.0001
Medication use					
Anxiolytics	5756	(36.5)	2550	(64.7)	<0.0001
Antipsychotics	1559	(9.9)	805	(20.4)	<0.0001
Antiepileptics	1547	(9.8)	773	(19.6)	<0.0001
Antidepressants	1656	(10.5)	743	(18.9)	<0.0001
Oral steroids	2549	(16.2)	672	(17.1)	0.1806

COPD = chronic obstructive pulmonary disease.

For peer review only

Table 2 Risk of fracture events for cohorts with and without liver cirrhosis[3]

	People without liver cirrhosis				People with liver cirrhosis				Risk of fracture
	n	Person-years	Events	Incidence[1]	n	Person-years	Events	Incidence[1]	HR (95% CI)[2]
All	15764	95430	1641	17.2	3941	23221	675	29.1	1.83 (1.67-2.01)
Female	4980	29405	718	24.4	1245	7352	249	33.9	1.53 (1.32-1.78)
Male	10784	66025	923	14.0	2696	15869	426	26.8	2.04 (1.81-2.31)
Age, 20-39 years	2544	15955	183	11.5	636	3838	97	25.3	2.12 (1.59-2.81)
Age, 40-49 years	3628	22574	295	13.1	907	5601	142	25.4	2.04 (1.63-2.54)
Age, 50-59 years	3624	21828	332	15.2	906	5442	124	22.8	1.67 (1.34-2.07)
Age, 60-69 years	3320	20111	373	18.5	830	4903	144	29.4	1.69 (1.38-2.06)
Age, ≥70 years	2648	14963	458	30.6	662	3437	168	48.9	1.70 (1.41-2.03)

CI = confidence intervals; HR = hazard ratio.

[1]Per 1000 person-years.

[2]People with liver cirrhosis vs. people without liver cirrhosis; Cox proportional hazard model with adjustment for all covariates in Table 1.

[3]In the subgroup analysis, the HRs of traumatic brain injury, neck or trunk fracture, fracture of upper limb, fracture of lower limb, and hip fracture associated with liver cirrhosis were 2.28 (95% CI 1.66-3.14), 1.75 (95% CI 1.44-2.12), 1.78 (95% CI 1.52-2.08), 1.94 (95% CI 1.65-2.27), and 2.22 (95% CI 1.70-2.89), respectively.

For peer review only

For peer review only - http://bmjopen.bmj.com/site/about/guidelines.xhtml

Table 3 Characteristics of fracture patients with and without liver cirrhosis

	Liver cirrhosis				
	No (N=680706)		Yes (N=7584)		*P*
Age, years	n	(%)	n	(%)	<0.0001
20-29	78164	(11.5)	82	(1.1)	
30-39	71008	(10.4)	718	(9.5)	
40-49	83951	(12.3)	1433	(18.9)	
50-59	110315	(16.2)	1400	(18.5)	
60-69	96376	(14.2)	1136	(15.0)	
≥70	240892	(35.4)	2815	(37.1)	
Sex					<0.0001
Female	330440	(48.5)	2586	(34.1)	
Male	350266	(51.5)	4998	(65.9)	
Low income	23243	(3.4)	607	(8.0)	<0.0001
Medical center	181324	(26.6)	1585	(20.9)	<0.0001
Coexisting medical conditions					
Mental disorders	102874	(15.1)	2027	(26.7)	<0.0001
Hypertension	135984	(20.0)	1604	(21.2)	0.0111
Diabetes	82560	(12.1)	1543	(20.4)	<0.0001
COPD	55169	(8.1)	876	(11.6)	<0.0001
Ischemic heart disease	47174	(6.9)	650	(8.6)	<0.0001
Stroke	26507	(3.9)	512	(6.8)	<0.0001
Congestive heart failure	13287	(2.0)	359	(4.7)	<0.0001
Parkinson's disease	17991	(2.6)	230	(3.0)	0.0355
Renal dialysis	7564	(1.1)	230	(3.0)	<0.0001
Hyperlipidemia	22779	(3.4)	120	(1.6)	<0.0001
Type of fracture					
Fracture with surgery	482458	(70.9)	4040	(53.3)	<0.0001
Traumatic brain injury	119165	(17.5)	1263	(16.7)	0.0520
Fracture of neck and trunk	97677	(14.4)	1500	(19.8)	<0.0001
Fracture of upper limb	256356	(37.7)	2285	(30.1)	<0.0001
Fracture of lower limb	322161	(47.3)	3807	(50.2)	<0.0001

COPD = chronic obstructive pulmonary disease.

For peer review only

Table 4 Adverse events after fracture in patients with liver cirrhosis

	No LC, %	LC, %	OR (95% CI)*
30-day in-hospital mortality	1.2	2.2	1.64 (1.40-1.93)
Septicemia	2.5	5.5	1.76 (1.59-1.95)
Acute renal failure	0.7	1.4	1.67 (1.37-2.04)
Medical expenditure, USD†	2212±2550	2500±2743	p<0.0001
Length of hospital stay, days†	8.5±13.8	9.6±10.5	p<0.0001

CI = confidence interval; LC = liver cirrhosis; OR = odds ratio.

*Adjusted for age, sex, low income, medical center, coexisting medical conditions and types of fracture.

†Mean±SD

For peer review only

Table 5 Liver cirrhosis associated with post-fracture adverse events in the stratification analysis by age, sex, and type of fracture

	People without liver cirrhosis			People with liver cirrhosis			Risk of events*
	n	Events*	Incidence	n	Events*	Incidence	OR (95% CI)†
Female	330440	11082	3.4	2586	219	8.5	2.01 (1.75-2.32)
Male	350266	15863	4.5	4998	402	8.0	1.62 (1.46-1.80)
Age, 20-29 years	78164	1862	2.4	82	6	7.3	2.21 (0.94-5.21)
Age, 30-39 years	71008	1698	2.4	718	47	6.6	1.82 (1.34-2.49)
Age, 40-49 years	83951	2215	2.6	1433	118	8.2	2.25 (1.83-2.75)
Age, 50-59 years	110315	3053	2.8	1400	99	7.1	1.89 (1.53-2.34)
Age, 60-69 years	96376	3362	3.5	1136	92	8.1	1.99 (1.59-2.48)
Age, ≥70 years	240892	14755	6.1	2815	259	9.2	1.42 (1.25-1.62)
Fracture with surgery	482458	12709	2.6	4040	297	7.4	2.17 (1.92-2.45)
Traumatic brain injury	119165	8617	7.2	1263	157	12.4	1.60 (1.35-1.90)
Neck and trunk fracture	97677	4864	5.0	1500	107	7.1	1.42 (1.16-1.73)
Upper limb fracture	256356	4706	1.8	2285	104	4.6	1.77 (1.44-2.17)
Lower limb fracture	322161	14467	4.5	3807	351	9.2	1.80 (1.61-2.02)

CI = confidence interval; OR = odds ratio.

*Any adverse events included 30-day in-hospital mortality, septicemia, and acute renal failure.

†Adjusted for all covariates in Table 3.

For peer review only - http://bmjopen.bmj.com/site/about/guidelines.xhtml

Table 6 Cirrhosis-related clinical indicators' effect after fracture outcomes for patients with liver cirrhosis

		30-day in-hospital adverse events*		
	n	events	incidence, %	OR (95% CI)†
No LC	680706	26945	4.0	1.00 (reference)
Effects of ADS				
LC without ADS	6397	530	8.3	1.71 (1.56-1.88)
LC with ADS	1187	91	7.7	1.96 (1.57-2.44)
Effects of jaundice				
LC without jaundice	7013	554	7.9	1.65 (1.51-1.81)
LC with jaundice	571	67	11.7	3.17 (2.44-4.13)
Effects of ascites				
LC without ascites	6337	479	7.6	1.60 (1.45-1.76)
LC with ascites	1247	142	11.4	2.52 (2.10-3.01)
Effects of GI hemorrhage				
LC without GI hemorrhage	5688	439	7.7	1.63 (1.48-1.80)
LC with GI hemorrhage	1896	182	9.6	2.10 (1.80-2.46)
Effects of hepatic coma				
LC without hepatic coma	6587	503	7.6	1.60 (1.46-1.76)
LC with hepatic coma	997	118	11.8	2.80 (2.30-3.41)
Number of cirrhotic indicators‡				
0	3772	254	6.7	1.37 (1.20-1.56)
1	2378	206	8.7	1.84 (1.59-2.13)
2	944	105	11.1	2.67 (2.17-3.30)
3	359	43	12.0	2.98 (2.15-4.14)
≥4	131	13	9.9	2.87 (1.60-5.15)

ADS, alcohol dependence syndrome; CI, confidence interval; GI, gastrointestinal; LC, liver cirrhosis; OR, odds ratio.

*Any adverse events included with 30-day in-hospital mortality, septicemia, acute renal failure.

†Adjusted for all covariates in Table 3.

‡Liver-related illnesses include alcohol dependence syndrome, ascites, jaundice, gastrointestinal hemorrhage, and hepatic coma.

For peer review only

STROBE 2007 (v4) checklist of items to be included in reports of observational studies in epidemiology*
Checklist for cohort, case-control, and cross-sectional studies (combined)

Section/Topic	Item #	Recommendation	Reported on page #
**Title and abstract**	1	(*a*) Indicate the study's design with a commonly used term in the title or the abstract	1, 3
		(*b*) Provide in the abstract an informative and balanced summary of what was done and what was found	3
**Introduction**			
Background/rationale	2	Explain the scientific background and rationale for the investigation being reported	4
Objectives	3	State specific objectives, including any pre-specified hypotheses	4, 5
**Methods**			
Study design	4	Present key elements of study design early in the paper	6
Setting	5	Describe the setting, locations, and relevant dates, including periods of recruitment, exposure, follow-up, and data collection	6, 7
Participants	6	(*a*) *Cohort study*—Give the eligibility criteria, and the sources and methods of selection of participants. Describe methods of follow-up<br>*Case-control study*—Give the eligibility criteria, and the sources and methods of case ascertainment and control selection. Give the rationale for the choice of cases and controls<br>*Cross-sectional study*—Give the eligibility criteria, and the sources and methods of selection of participants	6, 7
		(*b*) *Cohort study*—For matched studies, give matching criteria and number of exposed and unexposed<br>*Case-control study*—For matched studies, give matching criteria and the number of controls per case	6, 7
Variables	7	Clearly define all outcomes, exposures, predictors, potential confounders, and effect modifiers. Give diagnostic criteria, if applicable	7, 8, 9
Data sources/ measurement	8*	For each variable of interest, give sources of data and details of methods of assessment (measurement). Describe comparability of assessment methods if there is more than one group	7, 8, 9
Bias	9	Describe any efforts to address potential sources of bias	7, 8, 9
Study size	10	Explain how the study size was arrived at	7, 8, 9
Quantitative variables	11	Explain how quantitative variables were handled in the analyses. If applicable, describe which groupings were chosen and why	7, 8, 9
Statistical methods	12	(*a*) Describe all statistical methods, including those used to control for confounding	7, 8, 9
		(*b*) Describe any methods used to examine subgroups and interactions	7, 8, 9
		(*c*) Explain how missing data were addressed	7, 8, 9
		(*d*) *Cohort study*—If applicable, explain how loss to follow-up was addressed<br>*Case-control study*—If applicable, explain how matching of cases and controls was addressed	7, 8, 9

For peer review only

		Cross-sectional study—If applicable, describe analytical methods taking account of sampling strategy	
		(*e*) Describe any sensitivity analyses	7, 8, 9
**Results**			
Participants	13*	(a) Report numbers of individuals at each stage of study—eg numbers potentially eligible, examined for eligibility, confirmed eligible, included in the study, completing follow-up, and analysed	10, 11, 12
		(b) Give reasons for non-participation at each stage	10, 11, 12
		(c) Consider use of a flow diagram	10, 11, 12
Descriptive data	14*	(a) Give characteristics of study participants (eg demographic, clinical, social) and information on exposures and potential confounders	10, 11, 12
		(b) Indicate number of participants with missing data for each variable of interest	10, 11, 12
		(c) *Cohort study*—Summarise follow-up time (eg, average and total amount)	10, 11, 12
Outcome data	15*	*Cohort study*—Report numbers of outcome events or summary measures over time	10, 11, 12
		Case-control study—Report numbers in each exposure category, or summary measures of exposure	10, 11, 12
		Cross-sectional study—Report numbers of outcome events or summary measures	10, 11, 12
Main results	16	(*a*) Give unadjusted estimates and, if applicable, confounder-adjusted estimates and their precision (eg, 95% confidence interval). Make clear which confounders were adjusted for and why they were included	10, 11, 12
		(*b*) Report category boundaries when continuous variables were categorized	10, 11, 12
		(*c*) If relevant, consider translating estimates of relative risk into absolute risk for a meaningful time period	10, 11, 12
Other analyses	17	Report other analyses done—eg analyses of subgroups and interactions, and sensitivity analyses	10, 11, 12
**Discussion**			
Key results	18	Summarise key results with reference to study objectives	13
Limitations	19	Discuss limitations of the study, taking into account sources of potential bias or imprecision. Discuss both direction and magnitude of any potential bias	16
Interpretation	20	Give a cautious overall interpretation of results considering objectives, limitations, multiplicity of analyses, results from similar studies, and other relevant evidence	13-16
Generalisability	21	Discuss the generalisability (external validity) of the study results	13-16
**Other information**			
Funding	22	Give the source of funding and the role of the funders for the present study and, if applicable, for the original study on which the present article is based	23

*Give information separately for cases and controls in case-control studies and, if applicable, for exposed and unexposed groups in cohort and cross-sectional studies.

Note: An Explanation and Elaboration article discusses each checklist item and gives methodological background and published examples of transparent reporting. The STROBE checklist is best used in conjunction with this article (freely available on the Web sites of PLoS Medicine at http://www.plosmedicine.org/, Annals of Internal Medicine at http://www.annals.org/, and Epidemiology at http://www.epidem.com/). Information on the STROBE Initiative is available at www.strobe-statement.org.

For peer review only

Risk and adverse outcomes of fractures in patients with liver cirrhosis: two nationwide retrospective cohort studies

Journal:	*BMJ Open*
Manuscript ID	bmjopen-2017-017342.R1
Article Type:	Research
Date Submitted by the Author:	27-Jul-2017
Complete List of Authors:	Chen, Ta-Liang; Taipei Medical University Hospital, Department of Anesthesiology<br>Lin, Chao-Shun; Taipei Medical University Hospital, Department of Anesthesiology<br>Shih, Chun-Chuan; I-Shou University, School of Chinese Medicine for Post-Baccalaureate<br>Huang, Yu-Feng; Mackay Memorial Hospital Taitung Branch Hospital, Department of Anesthesiology<br>Yeh, Chun-Chieh; University of Illinois, Department of Surgery<br>Wu, Chih-Hsing; National Cheng Kung University Hospital, Family Medicine<br>Cherng, Yih-Giun; Shuang Ho Hospital, Taipei Medical University, Department of Anesthesiology<br>Liao, Chien-Chang; Taipei Medical University Hospital, Department of Anesthesiology; Taipei Medical University, Department of Anesthesiology, School of Medicine
**Primary Subject Heading**:	Epidemiology
Secondary Subject Heading:	Gastroenterology and hepatology, Rehabilitation medicine
Keywords:	liver cirrhosis, fracture, risk, outcome

SCHOLARONE™ Manuscripts

Risk and adverse outcomes of fractures in patients with liver cirrhosis: two nationwide retrospective cohort studies

Ta-Liang Chen,[1,2,3] Chao-Shun Lin,[1,2,3] Chun-Chuan Shih,[4] Yu-Feng Huang,[5] Chun-Chieh Yeh,[6,7] Chih-Hsing Wu,[8] Yih-Giun Cherng,[3,9] Chien-Chang Liao[1,2,3,9,10]

[1]Department of Anesthesiology, Taipei Medical University Hospital, Taipei, Taiwan

[2]Anesthesiology and Health Policy Research Center, Taipei Medical University Hospital, Taipei, Taiwan

[3]Department of Anesthesiology, School of Medicine, College of Medicine, Taipei Medical University, Taipei, Taiwan

[4]The School of Chinese Medicine for Post-Baccalaureate, I-Shou University, Kaohsiung, Taiwan

[5]Department of Anesthesiology, Taitung Mackay Memorial Hospital, Taitung, Taiwan

[6]Department of Surgery, China Medical University Hospital, Taichung, Taiwan

[7]Department of Surgery, University of Illinois, Chicago, United States of America

[8]Department of Family Medicine, National Cheng Kung University Hospital, Tainan, Taiwan

[9]Department of Anesthesiology, Shuan Ho Hospital, Taipei Medical University, New Taipei City, Taiwan

[10]School of Chinese Medicine, College of Chinese Medicine, China Medical University, Taichung, Taiwan

For peer review only

Correspondence:

Chien-Chang Liao, PhD, MPH

Associate Professor

Department of Anesthesiology, Taipei Medical University Hospital

252 Wuxing St., Taipei 11031, Taiwan

Tel: 886-2-2737-2181, ext. 8310

Fax: 886-2-2736-7344

E-mail: jacky48863027@yahoo.com.tw; ccliao@tmu.edu.tw

Abstract: 236 words; Text: 2243 words; References: 39

Running head: Liver cirrhosis and fracture

Abbreviations: CI=confidence interval; HR=hazard ratio; ICD-9-CM=International Classification of Diseases, Ninth Revision, Clinical Modification; LC=liver cirrhosis; OR=odds ratio

Key Words: liver cirrhosis; fracture; risk; outcomes

For peer review only

Abstract

Objective: The aim of this study is to evaluate fracture risk and post-fracture outcomes in patients with and without liver cirrhosis (LC).

Design: Retrospective cohort study and nested fracture cohort study.

Setting: This study was based on Taiwan's National Health Insurance Research Database that included information on: (1) 3941patients with aged 20 years and older newly diagnosed with LC between 2000 and 2003; (2) 688,560 hospitalized fracture patients aged 20 years and older between 2006 and 2013.

Primary and secondary outcome measures: Followed-up events of fracture from 2000 until 2008 were noted from medical claims to evaluate adjusted hazard ratios (HRs) and 95% confidence intervals (CIs) of fracture associated with LC. Adjusted odds ratios (ORs) and 95% CIs of adverse events after fracture were compared among patients with and without LC

Results: The incidences of fracture for people with and without LC were 28.0 and 16.9 per 1000 person-years, respectively. Compared with control, the adjusted HR of fracture was 1.71 (95% CI 1.55-1.87) for LC patients. Previous LC was associated with risks of septicemia (OR 1.87, 95 % CI 1.68-2.0), acute renal failure (OR 1.77, 95% CI 1.43-2.18), and 30-day in-hospital mortality (OR 1.71, 95 % CI 1.45-2.01) after fracture.

Conclusion: LC was associated with higher risk of fracture; LC patients in particular had more complications and 30-day in-hospital mortality after fracture. Fracture prevention and

For peer review only

attention to post-fracture adverse events are needed for these susceptible populations.

For peer review only

For peer review only - http://bmjopen.bmj.com/site/about/guidelines.xhtml

Strengths of this study

(1) Our study is a longitudinal retrospective cohort with large sample size.

(2) We used frequency matching and multivariate Cox proportional hazard models to control the confounding bias.

(3) This is the first study with comprehensive assessment of the impact of liver cirrhosis on the fracture risk and outcomes.

Limitations of this study

(1) Our data lack of clinical risk scores, lesion characteristics, biochemical measures, and lifestyles of fracture patients and cirrhosis patients.

(2) The detailed lifestyle included smoking, alcohol drinking, and physical activity were not available.

For peer review only

INTRODUCTION

Liver cirrhosis (LC) is the fourth-most-common cause of death in Europe, and causes more than one million deaths every year worldwide.[1,2] Prevalence of LC increased 68% during the 1992-2001 decade in the UK; in the US, it remains a pandemic chronic disease with more than 600,000 patients in 1999-2010 whose economic burden was shown to have doubled during 1998 and 2003.[3-5] Although the epidemiology, pathogenesis, prevention, and treatment of LC have been studied,[6] complications of liver cirrhosis are not well understood.

Fracture causes disability and mortality, and places economic burdens on societies.[7,8] Since many studies found patients with LC had increased risk of osteoporosis,[9-11] fall and fracture were considered complications for patients with LC.[12-20] However, these studies did not compare the risk of fracture or falls between people with and without LC. Small sample size,[12,14-18] inadequate control for confounding factors,[12-18] case-control study design,[12,14,16-19] focus on specific population,[12] lack of control group,[14,16,18,19] and of subgroup analysis [14-17,19,20] limited previous investigations. Information also was lacking on whether LC is associated with post-fracture adverse outcomes.

With the use of reimbursement claims from Taiwan's National Health Insurance program, we conducted two nationwide cohort studies. The retrospective cohort study seeks to validate the risk of fracture in patients with LC. Whether LC was associated with adverse outcomes

For peer review only - http://bmjopen.bmj.com/site/about/guidelines.xhtml

after fracture was reported in the nested fracture cohort study.

For peer review only

For peer review only - http://bmjopen.bmj.com/site/about/guidelines.xhtml

METHODS

Source of data

This study used the claims data of Taiwan's National Health Insurance Program, which was implemented in March 1995 and covers 99% of 23 million people nationwide. The National Health Research Institutes (NHRI) established the National Health Insurance Research Database to record all beneficiaries' information about inpatient and outpatient medical services. This includes basic patient demographics, physician's primary and secondary diagnoses, treatment procedures, prescribed medications, and medical expenditures. All contracted medical clinics, hospitals and medical centers are required to submit computerized claim documents for medical expenses. The validity of this database has been favorably evaluated, and research articles based on it have been accepted in scientific journals worldwide.[21-23]

Ethical approval

Insurance reimbursement claims used in this study were decoded with patient identifications scrambled for further research access. This study was conducted in accordance with the Helsinki Declaration. Although NHRI regulations do not require informed consent because patient identification has been decoded for privacy, this study also was approved by Taiwan's National Health Research Institutes (NHIRD-103-121) and Taipei Medical University's Joint Institutional Review Board (TMU-JIRB-201705063;

For peer review only - http://bmjopen.bmj.com/site/about/guidelines.xhtml

TMU-JIRB-201705084).

Study design

This investigation included two studies. In Study I (the retrospective cohort study), our purpose is to evaluate the risk of fracture for people with and without LC. From the representative sample of 1,000,000 insurance enrollees, we required at least two visits for medical care with the physician's primary diagnosis of cirrhosis of the liver to identify a cohort of 3941 newly diagnosed adults aged ≥ 20 years in 2000-2003. Those with only one medical visit with physician's diagnosis of liver cirrhosis were not considered as cirrhotic cases in this study. The frequency-matching procedure (by age and sex) was used to select the cohort, who had no previous medical records of LC. Both LC and non-LC cohorts had no history of fracture between the index date (date of LC diagnosis) and January 1, 1996 (the starting date of the Taiwan's National Health Insurance Program). That is to say, there was no recorded previous fracture from onset of database (1996) until the date of enrollment to the study (2000-2003). The outcome of this retrospective cohort study is incident event of fracture that was identified during the follow-up period from the index date until the end of 2008 for LC and non-LC cohorts.

In Study II (the nested fracture cohort study), our purpose is to evaluate the outcomes after fracture in fracture patients with and without history of LC. The study II included 688,560 hospitalized fracture patients in 2004-2013 and we identified 7854 with history of LC

For peer review only - http://bmjopen.bmj.com/site/about/guidelines.xhtml

(defined as at least two visits for medical care with the physician's primary diagnosis of liver cirrhosis) within pre-fracture 24 months. Thirty-day in-hospital mortality, septicemia, and acute renal failure after fracture were considered as post-fracture outcomes and compared between fracture patients with and without LC in the nested fracture cohort study.

Measurements and definitions

The variables in Study I and Study II were defined and described as followings. Patients' age was calculated by the date of fracture admission. Low-income status was determined by the National Health Insurance Bureau, which validates those qualified for waived health-care co-payment. Following previous suggestions,[21,22] fracture-associated medications were also analyzed; these included anxiolytics, antiepileptics, antipsychotics, antidepressants, and oral steroids. We used the *International Classification of Diseases, Ninth Revision, Clinical Modification* (ICD-9-CM) and administration codes of Taiwan's National Health Insurance to examine the physician's diagnoses and medical services. The coexisting medical conditions were calculated within 24 months before fracture admission. We identified patients' medical conditions and complications such as mental disorders (ICD-9-CM 290-319), hypertension (ICD-9-CM 410-405), chronic obstructive pulmonary disease (ICD-9-CM 490-496), diabetes (ICD-9-CM 250), ischemic heart disease (ICD-9-CM 410-414), stroke (ICD-9-CM 430-438), hyperlipidemia (ICD-9-CM 272.0, 272.1, and 272.2), heart failure (ICD-9-CM 428), renal dialysis (D8, D9), Parkinson's disease (ICD-9-CM 332), liver cirrhosis (ICD-9-CM 571.2,

For peer review only - http://bmjopen.bmj.com/site/about/guidelines.xhtml

571.5, and 571.6), fracture (ICD-9-CM 800-829), sepsis (ICD-9-CM 038 and 998.5), and acute renal failure (ICD-9-CM 548). Types of fracture included traumatic brain injury (ICD-9-CM 800-804), fracture of neck and trunk (ICD-9-CM 805-809), fracture of upper limb (ICD-9-CM 810-819), and fracture of lower limb (ICD-9-CM 820-829) were also identified. Thirty-day in-hospital mortality is the main outcome in the nested fracture cohort study. In the Study I, we identified co-existing medical conditions and medications in the baseline (before the enrollment date within 2 years) and follow-up period. In the Study II, we identified co-existing medical conditions and medications before fracture admission within 2 years.

Statistical analysis

In Study I, the categorical data for cohorts with and without LC were analyzed by chi-square tests. The adjusted hazard ratios (HRs) and confidence intervals (CIs) of fracture risk associated with liver cirrhosis were calculated in the multiple Cox proportional hazard models with controlling for age, sex, low income, mental disorders, hypertension, chronic obstructive pulmonary disease, diabetes, ischemic heart disease, stroke, hyperlipidemia, congestive heart failure, renal dialysis, Parkinson’s disease, anxiolytics, antipsychotics, antiepileptics, antidepressants, and oral steroids, as were associations between LC and fracture risk in males, females, and every age group.

In Study II, we used chi-square tests to examine other sociodemographic factors and

For peer review only

medical conditions in hospitalized fracture patients with and without history of LC. The multiple logistic regressions were used to calculate adjusted odds ratios (ORs) and 95% CIs of 30-day in-hospital mortality, sepsis, and acute renal failure after fracture associated with history of LC with controlling for age, sex, low income, mental disorders, hypertension, diabetes, chronic obstructive pulmonary disease, ischemic heart disease, stroke, congestive heart failure, Parkinson's disease, renal dialysis, hyperlipidemia, and types of fracture.

For peer review only

RESULTS

In Study I, there was no significant difference in age and sex between cohorts with and without LC because we used frequency-matching procedure (Table 1). The LC cohort had more medical conditions than the non-LC cohort; these included mental disorders ($p<0.0001$), hypertension ($p=0.0046$), chronic obstructive pulmonary disease ($p<0.0001$), diabetes ($p<0.0001$), ischemic heart disease ($p<0.0001$), stroke ($p<0.0001$), hyperlipidemia ($p=0.0003$), heart failure ($p<0.0001$), renal dialysis ($p<0.0001$), and Parkinson's disease ($p<0.0001$). The use of anxiolytics ($p<0.0001$), antiepileptics ($p<0.0001$), antipsychotics ($p<0.0001$), and antidepressants ($p<0.0001$) was also higher in patients with LC than in people without LC.

During the 5-8 years of follow-up (Study I), the incidences of fracture for cohorts with and without LC were 29.1 and 17.2 per 1000 person-years, respectively (Table 2). The increased risk of fracture was found in LC cohort after adjustment (HR 1.83, 95% CI 1.67-2.01). We also found significant associations between fracture risk and LC in males (HR 2.04, 95% CI 1.81-2.31), females (HR 1.53, 95% CI 1.32-1.78), and people aged 20-39 (HR 2.12, 95% CI 1.59-2.81), 40-49 (HR 2.04, 95% CI 1.63-2.54), 50-59 (HR 1.67, 95% CI 1.34-2.07), 60-69 (HR 1.69, 95% CI 1.38-2.06), and ≥70 years (HR 1.70, 95% CI 1.41-2.03).

The Study II included 688,560 hospitalized fracture patients (Table 3). Fracture patients with LC had lower proportions of young adults ($p<0.0001$) but higher proportions of males ($p<0.0001$) and low-income status ($p<0.0001$). More history of mental disorders, diabetes,

For peer review only - http://bmjopen.bmj.com/site/about/guidelines.xhtml

chronic obstructive pulmonary disease, ischemic heart disease, stroke, congestive heart failure, Parkinson's disease, and renal dialysis were found in patients with LC than in control ($p<0.05$ for all). Fracture patients with previous LC had higher proportions of neck or trunk fracture ($p<0.0001$) and lower limb fracture ($p<0.0001$).

In Study II, higher risks for post-fracture sepsis (OR 1.76, 95% CI 1.59-1.95), acute renal failure (OR 1.67, 95% CI 1.37-2.04), and 30-day in-hospital mortality (OR 1.64, 95% CI 1.40-1.93) were associated with previous LC (Table 4). Fracture patients with LC had higher mean length of hospital stay (9.6±10.5 vs. 8.5±13.8 days, $p<0.0001$) and medical expenditure (2500±2743 vs. 2212±2550 USD, $p<0.0001$) than patients without LC.

In Study II (Table 5), previous LC was associated with higher risks of adverse events after fracture in females (OR 2.01, 95% CI 1.75-2.32), males (OR 1.62, 95% CI 1.46-1.80), and patients aged 30-39 (OR 1.82, 95% CI 1.34-2.49), 40-49 (OR 2.25, 95% CI 1.83-2.75), 50-59 (OR 1.89, 95% CI 1.53-2.34), 60-69 (OR 1.99, 95% CI 1.59-2.48), and ≥70 years (OR 1.42, 95% CI 1.25-1.62). Associations between post-fracture adverse events and LC were significant in patients with fracture receiving surgery (OR 2.17, 95% CI 1.92-2.45), traumatic brain injury (OR 1.60, 95% CI 1.35-1.90), or fracture of upper limb (OR 1.77, 95% CI 1.44-2.17), lower limb (OR 1.80, 95% CI 1.61-2.02), and neck or trunk (OR 1.42, 95% CI 1.16-1.73).

The Study II showed that alcohol dependence syndrome (OR 1.96, 95% CI 1.57-2.44),

For peer review only - http://bmjopen.bmj.com/site/about/guidelines.xhtml

jaundice (OR 3.17, 95% CI 2.44-4.13), ascites (OR 2.52, 95% CI 2.10-3.01), gastrointestinal hemorrhage (OR 2.10, 95% CI 1.80-2.46), and hepatic coma (OR 2.80, 95% CI 2.30-3.41) were significant determinants for post-fracture adverse events (Table 6). The risk of post-fracture adverse events increased with the number of cirrhotic indicators increased (OR 2.87, 95% CI 1.60-5.15).

For peer review only

For peer review only - http://bmjopen.bmj.com/site/about/guidelines.xhtml

DISCUSSION

In Study I (the retrospective cohort study), we observed a significant association between LC and fracture, with an 83% increased risk of fracture in patients with LC during the 5-8 years of follow-up. In Study II (the nested fracture cohort study), we observed that patients with LC had significantly higher post-fracture complications and 30-day in-hospital mortality. Clinical indicators of the severity of LC, such as alcohol dependence syndrome, jaundice, ascites, gastrointestinal hemorrhage, and hepatic coma were all associated with more post-fracture adverse events.

In general, the prevalence of LC was higher in males than in females.[4,24] Older age and low socioeconomic status were factors associated with higher risk of LC.[3,23,25] Older age, males, and low income were also risk factors for fracture, and were controlled by using the multivariate Cox proportional models to control these potential confounding effects for the analysis of association between LC and fracture risk.[26] Furthermore, we found that the association between LC and fracture risk remains significant in every age group and both sexes. The significant impact of LC on post-fracture adverse events was noted in males, females, various age, and people with various types of fracture. This phenomenon revealed the possible causal inference that LC was associated with fracture risk and post-fracture adverse events from the viewpoint of epidemiology. These findings were crucial because several previous studies were limited by focusing on specific population by failing to

For peer review only - http://bmjopen.bmj.com/site/about/guidelines.xhtml

investigate the association in subgroup analysis.[14-17,19,20]

Mental disorders, hypertension, chronic obstructive pulmonary disease, diabetes, ischemic heart disease, stroke, hyperlipidemia, congestive heart failure, renal dialysis and Parkinson's disease were considered as co-existing medical conditions that also were fracture risk factors.[21,22,27-29,30-32] Confounding bias may occurred in previous studies that lacked multivariate adjustment for these fracture-related and/or cirrhosis-related medical conditions.[12-18] Therefore, we used multiple Cox proportional hazard and multiple logistic regression models to control the confounding effects of medical conditions when investigating the risks and outcomes of fracture in patients with LC in Study I and Study II.

Unlike previous investigations, we studied the impact of LC on post-fracture outcomes such as septicemia, acute renal failure, and mortality.[12-20] Fracture patients with history of LC had longer hospital stay and medical expenditure than non-LC people in the nested fracture cohort study. Patients with LC had circulatory dysfunction and poor immune systems that compromise systemic inflammatory response and make them prone to renal failure and septicemia,[23,33] particularly those patients with cirrhotic indicators such as alcohol dependence syndrome, jaundice, ascites, gastrointestinal hemorrhage, and hepatic coma. Therefore, higher mortality and consumption of medical resources might be encountered in the LC population during fracture admissions.

Associations between LC and fracture risk suggest several possible explanations. First,

For peer review only - http://bmjopen.bmj.com/site/about/guidelines.xhtml

many studies found LC patients had increased risk of osteoporosis,[9-11] a condition that is an important determinant for fracture.[8] Fracture due to bone loss and the pathogenesis of osteoporosis among patients with LC is complex and multifactorial, and the exact mechanism remains uncertain. A previous study showed cirrhotic patients with osteoporosis had lower levels of insulin-like growth factor-1 than cirrhotic patients without osteoporosis.[34] Insulin-like growth factor-1 plays a major role in bone remodeling and maintenance of bone mass, and was found to be reduced in advanced cirrhosis.[35] Cirrhotic patients' hyperbilirubinemia also has been shown to impair osteoblast proliferation, resulting in decreased bone formation and possibly accounting for the increased risks of fracture.[36] Second, corticosteroids are frequently used in patients with autoimmune hepatitis and other inflammatory disorders. Even budesonide, a corticosteroid with minimal systemic availability, might lead to accelerated bone loss in cirrhotic patients and postmenopausal women.[37] We postulated that medications used in the treatment of LC could also have an adverse effect on bone and calcium mobilization and subsequent osteoporosis. Third, hepatic coma, poor cognitive function, and psychiatric illness may play roles in the association between LC and risk of fracture.[25,38] Though hepatic encephalopathy does not commonly occur in patients with LC, its contribution to falls should not be ignored.[39]

Some study limitations need to be addressed when interpreting the present results. First, this study used retrospective reimbursement claims, which lack data on severity of LC,

For peer review only - http://bmjopen.bmj.com/site/about/guidelines.xhtml

lifestyle factors, personal characteristics, and biochemical data. Compared with the previous well-adjustment study [19], the above unavailable information is an important source of bias. Second, since the patients were selected based on diagnoses from hospital inpatient care registers, patients with minor LC but no symptoms might not seek medical services, leading to underestimation of fracture risk in LC patients because some minor LC patients may have been in the non-LC group. Third, because our results are based on the data from Taiwan's National Health Insurance, findings of this study could not be directly generalized to other populations.

Conclusion

Our two cohort studies provide population-based evidence that LC is an important risk factor for fracture. We also noted that fracture patients with various clinical indicators of LC severity face increased risks of post-fracture adverse events. We demonstrated risk factor analysis and a variety of clinical suggestions, including prevention, risk assessment and outcome-related information to fracture patients with LC. Strategies to prevent fracture and meticulous care to reduce post-fracture adverse events should be routinely considered for this population.

For peer review only - http://bmjopen.bmj.com/site/about/guidelines.xhtml

REFERENCES

1. Lozano R, Naghavi M, Foreman K, Lim S, Shibuya K, Aboyans V, et al. Global and regional mortality from 235 causes of death for 20 age groups in 1990 and 2010: a systematic analysis for the Global Burden of Disease Study 2010. *Lancet.* 2012;380:2095-128.

2. Blachier M, Leleu H, Peck-Radosavljevic M, Valla DC, Roudot-Thoraval F. The burden of liver disease in Europe: a review of available epidemiological data. *J Hepatol.* 2013;58:593-608.

3. Scaglione S, Kliethermes S, Cao G, Shoham D, Durazo R, Luke A, et al. The epidemiology of cirrhosis in the United States: a population-based study. *J Clin Gastroenterol.* 2015;49:690-6.

4. Fleming KM, Aithal GP, Solaymani-Dodaran M, Card TR, West J. Incidence and prevalence of cirrhosis in the United Kingdom, 1992-2001: a general population-based study. *J Hepatol.* 2008;49:732-8.

5. Nguyen GC, Segev DL, Thuluvath PJ. Nationwide increase in hospitalizations and hepatitis C among inpatients with cirrhosis and sequelae of portal hypertension. *Clin Gastroenterol Hepatol.* 2007;5:1092-9.

6. Tsochatzis EA, Bosch J, Burroughs AK. Liver cirrhosis. *Lancet.* 2014;383:1749-61.

7. Bliuc D, Nguyen ND, Milch VE, Nguyen TV, Eisman JA, Center JR. Mortality risk

For peer review only

For peer review only - http://bmjopen.bmj.com/site/about/guidelines.xhtml

associated with low-trauma osteoporotic fracture and subsequent fracture in men and women. *JAMA.* 2009;301:513-21.

8. Burge R, Dawson-Hughes B, Solomon DH, Wong JB, King A, Tosteson A. Incidence and economic burden of osteoporosis-related fractures in the United States, 2005-2025. *J Bone Miner Res.* 2007;22:465-75.
9. Lupoli R, Di Minno A, Spadarella G, Ambrosino P, Panico A, Tarantino L, et al. The risk of osteoporosis in patients with liver cirrhosis: a meta-analysis of literature studies. *Clin Endocrinol.* 2016;84:30-8.
10. Guañabens N, Parés A, Ros I, Caballería L, Pons F, Vidal S, et al. Severity of cholestasis and advanced histological stage but not menopausal status are the major risk factors for osteoporosis in primary biliary cirrhosis. *J Hepatol.* 2005;42:573-7.
11. Mounach A, Ouzzif Z, Wariaghli G, Achemlal L, Benbaghdadi I, Aouragh A, et al. Primary biliary cirrhosis and osteoporosis: a case-control study. *J Bone Miner Metab.* 2008;26:379-84.
12. Boulton-Jones JR, Fenn RM, West J, Logan RF, Ryder SD. Fracture risk of women with primary biliary cirrhosis: no increase compared with general population controls. *Aliment Pharmacol Ther.* 2004;20:551-7.
13. Bang UC, Benfield T, Bendtsen F, Hyldstrup L, Beck Jensen JE. The risk of fractures among patients with cirrhosis or chronic pancreatitis. *Clin Gastroenterol Hepatol.*

For peer review only

2014;12:320-6.

14. Román E1, Córdoba J, Torrens M, Guarner C, Soriano G. Falls and cognitive dysfunction impair health-related quality of life in patients with cirrhosis. *Eur J Gastroenterol Hepatol.* 2013;25:77-84.

15. Solaymani-Dodaran M, Card TR, Aithal GP, West J. Fracture risk in people with primary biliary cirrhosis: a population-based cohort study. *Gastroenterology.* 2006;131:1752-7.

16. Guañabens N, Cerdá D, Monegal A, Pons F, Caballería L, Peris P, et al. Low bone mass and severity of cholestasis affect fracture risk in patients with primary biliary cirrhosis. *Gastroenterology.* 2010;138:2348-56.

17. Frith J, Kerr S, Robinson L, Elliott C, Ghazala C, Wilton K, et al. Primary biliary cirrhosis is associated with falls and significant fall related injury. *QJM.* 2010;103:153-61.

18. Soriano G, Román E, Córdoba J, Torrens M, Poca M, Torras X, et al. Cognitive dysfunction in cirrhosis is associated with falls: a prospective study. *Hepatology.* 2012;55:1922-30.

19. Tapper EB, Risech-Neyman Y, Sengupta N. Psychoactive medications increase the risk of falls and fall-related injuries in hospitalized patients with cirrhosis. *Clin Gastroenterol Hepatol.* 2015;13:1670-5.

20. Tsai CF, Liu CJ, Chen TJ, Chu CJ, Lin HC, Lee FY, et al. Increased incidence of orthopedic fractures in cirrhotic patients: a nationwide population-based study. *J Hepatol.*

For peer review only

2013;58:706-14.

21. Huang YF, Cherng YG, Hsu SP, Yeh CC, Chou YC, Wu CH, et al. Risk and adverse outcomes of fractures in patients with Parkinson's disease: two nationwide studies. *Osteoporos Int.* 2015;26:1723-32.

22. Liao CC, Lin CS, Shih CC, Yeh CC, Chang YC, Lee YW, et al. Increased risk of fracture and postfracture adverse events in patients with diabetes: two nationwide population-based retrospective cohort studies. *Diabetes Care.* 2014;37:2246-52.

23. Lin CS, Lin SY, Chang CC, Wang HH, Liao CC, Chen TL. Postoperative adverse outcomes after non-hepatic surgery in patients with liver cirrhosis. *Br J Surg.* 2013;100:1784-90.

24. Scaglione S, Kliethermes S, Cao G, Shoham D, Durazo R, Luke A, et al. The epidemiology of cirrhosis in the United States: a population-based study. *J Clin Gastroenterol.* 2015;49:690-6.

25. Bajaj JS, Riggio O, Allampati S, Prakash R, Gioia S, Onori E, et al. Cognitive dysfunction is associated with poor socioeconomic status in patients with cirrhosis: an international multicenter study. *Clin Gastroenterol Hepatol.* 2013;11:1511-6.

26. Trimpou P, Landin-Wilhelmsen K, Odén A, Rosengren A, Wilhelmsen L. Male risk factors for hip fracture-a 30-year follow-up study in 7,495 men. *Osteoporos Int.* 2010;21:409-16.

27. Jepsen P, Vilstrup H, Lash TL. Development and validation of a comorbidity scoring

For peer review only

system for patients with cirrhosis. *Gastroenterology.* 2014;146:147-56.

28. Gershwin ME, Selmi C, Worman HJ, Gold EB, Watnik M, Utts J, et al. Risk factors and comorbidities in primary biliary cirrhosis: a controlled interview-based study of 1032 patients. *Hepatology.* 2005;42:1194-202.

29. Tryc AB, Goldbecker A, Berding G, Rümke S, Afshar K, Shahrezaei GH, et al. Cirrhosis-related Parkinsonism: prevalence, mechanisms and response to treatments. *J Hepatol.* 2013;58:698-705.

30. Dennison EM, Compston JE, Flahive J, Siris ES, Gehlbach SH, Adachi JD, et al. Effect of co-morbidities on fracture risk: findings from the Global Longitudinal Study of Osteoporosis in Women (GLOW). *Bone.* 2012;50:1288-93.

31. Hippisley-Cox J, Coupland C. Derivation and validation of updated QFracture algorithm to predict risk of osteoporotic fracture in primary care in the United Kingdom: prospective open cohort study. *BMJ.* 2012;344:e3427.

32. Dennison EM, Compston JE, Flahive J, Siris ES, Gehlbach SH, Adachi JD, et al. Effect of co-morbidities on fracture risk: findings from the Global Longitudinal Study of Osteoporosis in Women (GLOW). *Bone.* 2012;50:1288-93.

33. Gines P, Schrier RW. Renal failure in cirrhosis. *N Engl J Med.* 2009;361:1279-90.

34. Gallego-Rojo FJ, Gonzalez-Calvin JL, Muñoz-Torres M, Mundi JL, Fernandez-Perez R, Rodrigo-Moreno D. Bone mineral density, serum insulin-like growth factor I, and bone

For peer review only

For peer review only - http://bmjopen.bmj.com/site/about/guidelines.xhtml

turnover markers in viral cirrhosis. *Hepatology.* 1998;28:695-9.

35. Leslie WD, Bernstein CN, Leboff MS. AGA technical review on osteoporosis in hepatic disorders. *Gastroenterology.* 2003;125:941-66.

36. Janes CH, Dickson ER, Okazaki R, Bonde S, McDonagh AF, Riggs BL. Role of hyperbilirubinemia in the impairment of osteoblast proliferation associated with cholestatic jaundice. *J Clin Invest.* 1995;95:2581-6.

37. Angulo P, Jorgensen RA, Keach JC, Dickson ER, Smith C, Lindor KD. Oral budesonide in the treatment of patients with primary biliary cirrhosis with a suboptimal response to ursodeoxycholic acid. *Hepatology.* 2000;31:318-23.

38. Van Os E, van den Broek WW, Mulder PG, ter Borg PC, Bruijn JA, van Buuren HR. Depression in patients with primary biliary cirrhosis and primary sclerosing cholangitis. *J Hepatol.* 2007;46:1099-103.

39. Román E, Córdoba J, Torrens M, Torras X, Villanueva C, Vargas V, et al. Minimal hepatic encephalopathy is associated with falls. *Am J Gastroenterol.* 2011;106:476-82.

For peer review only

Acknowledgments: This study is based in part on data obtained from the National Health Insurance Research Database provided by the Bureau of National Health Insurance, Ministry of Health and Welfare, and managed by the National Health Research Institutes. The interpretation and conclusions contained herein do not represent those of the Bureau of National Health Insurance, Ministry of Health and Welfare, or National Health Research Institutes.

Competing interests: None.

Financial support: This research was supported in part by Shuang Ho Hospital, Taipei Medical University (104TMU-SHH-23), Taiwan's Ministry of Science and Technology (MOST105-2629-B-038-001; MOST105-2314-B-038-025; MOST104-2314-B-038-027-MY2; NSC102-2314-B-038-021-MY3), and Taiwan's Ministry of Health and Welfare Clinical Trial and Research Center of Excellence (MOHW105-TDU-B-212-133019).

Data sharing statement: dataset available from the Taiwan's National Health Research Institutes (http://nhird.nhri.org.tw/index1.php).

Author contribution: All the authors revised and approved the contents of the submitted article. TLC and CCL created the idea of the manuscript and wrote the draft. CCL conducted statistical analysis of data. All the authors made substantial contributions to interpretation of data and carried out a critical revision of the manuscript for important intellectual content.

For peer review only - http://bmjopen.bmj.com/site/about/guidelines.xhtml

For peer review only

Table 1 Sociodemographics, coexisting medical conditions, and medication use in people with and without liver cirrhosis

	Non-liver cirrhosis (N=15764)		Liver cirrhosis (N=3941)		*P* value
Sex	n	(%)	N	(%)	1.0000
Female	4980	(31.6)	1245	(31.6)	
Male	10784	(68.4)	2696	(68.4)	
Age, years					1.0000
20-29	620	(3.9)	155	(3.9)	
30-39	1924	(12.2)	481	(12.2)	
40-49	3628	(23.0)	907	(23.0)	
50-59	3624	(23.0)	906	(23.0)	
60-69	3320	(21.1)	830	(21.1)	
≥70	2648	(16.8)	662	(16.8)	
Low income	362	(2.3)	211	(5.3)	<0.0001
Coexisting medical conditions					
Mental disorders	3974	(25.2)	1437	(36.5)	<0.0001
Hypertension	5248	(33.3)	1406	(35.7)	0.0046
COPD	2892	(18.4)	992	(25.2)	<0.0001
Diabetes	2242	(14.2)	944	(24.0)	<0.0001
Ischemic heart disease	2623	(16.6)	788	(20.0)	<0.0001
Stroke	941	(6.0)	338	(8.6)	<0.0001
Hyperlipidemia	1468	(9.3)	294	(7.5)	0.0003
Congestive heart failure	453	(2.9)	255	(6.5)	<0.0001
Renal dialysis	123	(0.8)	112	(2.8)	<0.0001
Parkinson's disease	255	(1.6)	105	(2.7)	<0.0001
Medication use					
Anxiolytics	5756	(36.5)	2550	(64.7)	<0.0001
Antipsychotics	1559	(9.9)	805	(20.4)	<0.0001
Antiepileptics	1547	(9.8)	773	(19.6)	<0.0001
Antidepressants	1656	(10.5)	743	(18.9)	<0.0001
Oral steroids	2549	(16.2)	672	(17.1)	0.1806

COPD = chronic obstructive pulmonary disease.

For peer review only

For peer review only - http://bmjopen.bmj.com/site/about/guidelines.xhtml

Table 2 Risk of fracture events for cohorts with and without liver cirrhosis[3]

	People without liver cirrhosis				People with liver cirrhosis				Risk of fracture
	n	Person-years	Events	Incidence[1]	n	Person-years	Events	Incidence[1]	HR (95% CI)[2]
All	15764	95430	1641	17.2	3941	23221	675	29.1	1.83 (1.67-2.01)
Female	4980	29405	718	24.4	1245	7352	249	33.9	1.53 (1.32-1.78)
Male	10784	66025	923	14.0	2696	15869	426	26.8	2.04 (1.81-2.31)
Age, 20-39 years	2544	15955	183	11.5	636	3838	97	25.3	2.12 (1.59-2.81)
Age, 40-49 years	3628	22574	295	13.1	907	5601	142	25.4	2.04 (1.63-2.54)
Age, 50-59 years	3624	21828	332	15.2	906	5442	124	22.8	1.67 (1.34-2.07)
Age, 60-69 years	3320	20111	373	18.5	830	4903	144	29.4	1.69 (1.38-2.06)
Age, ≥70 years	2648	14963	458	30.6	662	3437	168	48.9	1.70 (1.41-2.03)

CI = confidence intervals; HR = hazard ratio.

[1]Per 1000 person-years.

[2]People with liver cirrhosis vs. people without liver cirrhosis; Cox proportional hazard model with controlling for all covariates listed in Table 1.

[3]In the subgroup analysis, the HRs of traumatic brain injury, neck or trunk fracture, fracture of upper limb, fracture of lower limb, and hip fracture associated with liver cirrhosis were 2.28 (95% CI 1.66-3.14), 1.75 (95% CI 1.44-2.12), 1.78 (95% CI 1.52-2.08), 1.94 (95% CI 1.65-2.27), and 2.22 (95% CI 1.70-2.89), respectively.

For peer review only

For peer review only - http://bmjopen.bmj.com/site/about/guidelines.xhtml

Table 3 Characteristics of fracture patients with and without liver cirrhosis

	Liver cirrhosis				
	No (N=680706)		Yes (N=7584)		*P*
Age, years	n	(%)	n	(%)	<0.0001
20-29	78164	(11.5)	82	(1.1)	
30-39	71008	(10.4)	718	(9.5)	
40-49	83951	(12.3)	1433	(18.9)	
50-59	110315	(16.2)	1400	(18.5)	
60-69	96376	(14.2)	1136	(15.0)	
≥70	240892	(35.4)	2815	(37.1)	
Sex					<0.0001
Female	330440	(48.5)	2586	(34.1)	
Male	350266	(51.5)	4998	(65.9)	
Low income	23243	(3.4)	607	(8.0)	<0.0001
Medical center	181324	(26.6)	1585	(20.9)	<0.0001
Coexisting medical conditions					
Mental disorders	102874	(15.1)	2027	(26.7)	<0.0001
Hypertension	135984	(20.0)	1604	(21.2)	0.0111
Diabetes	82560	(12.1)	1543	(20.4)	<0.0001
COPD	55169	(8.1)	876	(11.6)	<0.0001
Ischemic heart disease	47174	(6.9)	650	(8.6)	<0.0001
Stroke	26507	(3.9)	512	(6.8)	<0.0001
Congestive heart failure	13287	(2.0)	359	(4.7)	<0.0001
Parkinson's disease	17991	(2.6)	230	(3.0)	0.0355
Renal dialysis	7564	(1.1)	230	(3.0)	<0.0001
Hyperlipidemia	22779	(3.4)	120	(1.6)	<0.0001
Types of fracture					
Fracture with surgery	482458	(70.9)	4040	(53.3)	<0.0001
Traumatic brain injury	119165	(17.5)	1263	(16.7)	0.0520
Fracture of neck and trunk	97677	(14.4)	1500	(19.8)	<0.0001
Fracture of upper limb	256356	(37.7)	2285	(30.1)	<0.0001
Fracture of lower limb	322161	(47.3)	3807	(50.2)	<0.0001
Hip fracture	172592	(25.4)	2551	(33.6)	<0.0001
Open fractures	49783	(7.3)	432	(5.7)	<0.0001

COPD = chronic obstructive pulmonary disease.

For peer review only

For peer review only - http://bmjopen.bmj.com/site/about/guidelines.xhtml

Table 4 Adverse events after fracture in patients with and without liver cirrhosis

	No LC, %	LC, %	OR (95% CI)*
30-day in-hospital mortality	1.2	2.2	1.61 (1.37-1.89)
Septicemia	2.5	5.5	1.77 (1.60-1.96)
Acute renal failure	0.7	1.4	1.63 (1.33-1.99)
Medical expenditure, USD†	2212±2550	2500±2743	158 (105-211)
Length of hospital stay, days†	8.5±13.8	9.6±10.5	0.22 (0.08-0.52)

CI = confidence interval; LC = liver cirrhosis; OR = odds ratio.

*Controlled for all covariates listed in Table 3.

†t-test showed mean±SD, p<0.0001 in medical expenditure and length of hospital stay; beta coefficients and 95% CIs of medical expenditure and length of hospital stay associated with liver cirrhosis were calculated in multiple linear regression.

For peer review only

For peer review only - http://bmjopen.bmj.com/site/about/guidelines.xhtml

Table 5 Liver cirrhosis associated with post-fracture adverse events in the stratification analysis by age, sex, and type of fracture

	People without liver cirrhosis			People with liver cirrhosis			Risk of events*
	n	Events*	Incidence	n	Events*	Incidence	OR (95% CI)†
Female	330440	11082	3.4	2586	219	8.5	2.00 (1.73-2.30)
Male	350266	15863	4.5	4998	402	8.0	1.61 (1.45-1.80)
Age, 20-29 years	78164	1862	2.4	82	6	7.3	2.34 (1.00-5.52)
Age, 30-39 years	71008	1698	2.4	718	47	6.6	1.84 (1.35-2.51)
Age, 40-49 years	83951	2215	2.6	1433	118	8.2	2.29 (1.87-2.81)
Age, 50-59 years	110315	3053	2.8	1400	99	7.1	1.90 (1.53-2.36)
Age, 60-69 years	96376	3362	3.5	1136	92	8.1	1.99 (1.60-2.49)
Age, ≥70 years	240892	14755	6.1	2815	259	9.2	1.41 (1.24-1.61)
Fracture with surgery	482458	12709	2.6	4040	297	7.4	2.16 (1.91-2.44)
Traumatic brain injury	119165	8617	7.2	1263	157	12.4	1.60 (1.35-1.90)
Neck and trunk fracture	97677	4864	5.0	1500	107	7.1	1.42 (1.16-1.73)
Upper limb fracture	256356	4706	1.8	2285	104	4.6	1.78 (1.45-2.18)
Lower limb fracture	322161	14467	4.5	3807	351	9.2	1.79 (1.60-2.00)
Hip fracture	172592	10059	5.8	2551	265	10.4	1.71 (1.23-2.37)

CI = confidence interval; OR = odds ratio.

*Any adverse events included 30-day in-hospital mortality, septicemia, and acute renal failure.

†Controlled for all covariates listed in Table 3.

For peer review only

For peer review only - http://bmjopen.bmj.com/site/about/guidelines.xhtml

Table 6 Cirrhosis-related clinical indicators' effect on the outcomes of fracture admission in patients with liver cirrhosis

Characteristics of cirrhosis before fracture admission	n	30-day in-hospital adverse events* events	incidence, %	OR (95% CI)†
No LC	680706	26945	4.0	1.00 (reference)
Effects of liver admission				
LC without liver admission	5068	401	7.9	1.65 (1.49-1.84)
LC with liver admission	2516	220	8.7	1.91 (1.65-2.20)
Effects of ADS				
LC without ADS	6397	530	8.3	1.70 (1.55-1.86)
LC with ADS	1187	91	7.7	1.97 (1.58-2.45)
Effects of jaundice				
LC without jaundice	7013	554	7.9	1.64 (1.50-1.80)
LC with jaundice	571	67	11.7	3.15 (2.42-4.10)
Effects of ascites				
LC without ascites	6337	479	7.6	1.59 (1.45-1.75)
LC with ascites	1247	142	11.4	2.49 (2.08-2.98)
Effects of GI hemorrhage				
LC without GI hemorrhage	5688	439	7.7	1.62 (1.46-1.79)
LC with GI hemorrhage	1896	182	9.6	2.09 (1.79-2.45)
Effects of hepatic coma				
LC without hepatic coma	6587	503	7.6	1.59 (1.45-1.75)
LC with hepatic coma	997	118	11.8	2.77 (2.27-3.38)
Number of cirrhotic indicators‡				
0	3772	254	6.7	1.36 (1.19-1.54)
1	2378	206	8.7	1.83 (1.58-2.12)
2	944	105	11.1	2.65 (2.15-3.27)
3	359	43	12.0	2.95 (2.12-4.09)
≥4	131	13	9.9	2.94 (1.64-5.29)

ADS, alcohol dependence syndrome; CI, confidence interval; GI, gastrointestinal; LC, liver cirrhosis; OR, odds ratio.

*Adverse events included with 30-day in-hospital mortality, septicemia, and acute renal failure.

†Controlled for all covariates listed in Table 3.

‡Liver-related illnesses include alcohol dependence syndrome, ascites, jaundice, gastrointestinal hemorrhage, and hepatic coma.

For peer review only - http://bmjopen.bmj.com/site/about/guidelines.xhtml

STROBE 2007 (v4) checklist of items to be included in reports of observational studies in epidemiology*
Checklist for cohort, case-control, and cross-sectional studies (combined)

Section/Topic	Item #	Recommendation	Reported on page #
**Title and abstract**	1	(*a*) Indicate the study's design with a commonly used term in the title or the abstract	1, 3
		(*b*) Provide in the abstract an informative and balanced summary of what was done and what was found	3
**Introduction**			
Background/rationale	2	Explain the scientific background and rationale for the investigation being reported	4
Objectives	3	State specific objectives, including any pre-specified hypotheses	4, 5
**Methods**			
Study design	4	Present key elements of study design early in the paper	6
Setting	5	Describe the setting, locations, and relevant dates, including periods of recruitment, exposure, follow-up, and data collection	6, 7
Participants	6	(*a*) *Cohort study*—Give the eligibility criteria, and the sources and methods of selection of participants. Describe methods of follow-up<br>*Case-control study*—Give the eligibility criteria, and the sources and methods of case ascertainment and control selection. Give the rationale for the choice of cases and controls<br>*Cross-sectional study*—Give the eligibility criteria, and the sources and methods of selection of participants	6, 7
		(*b*) *Cohort study*—For matched studies, give matching criteria and number of exposed and unexposed<br>*Case-control study*—For matched studies, give matching criteria and the number of controls per case	6, 7
Variables	7	Clearly define all outcomes, exposures, predictors, potential confounders, and effect modifiers. Give diagnostic criteria, if applicable	7, 8, 9
Data sources/ measurement	8*	For each variable of interest, give sources of data and details of methods of assessment (measurement). Describe comparability of assessment methods if there is more than one group	7, 8, 9
Bias	9	Describe any efforts to address potential sources of bias	7, 8, 9
Study size	10	Explain how the study size was arrived at	7, 8, 9
Quantitative variables	11	Explain how quantitative variables were handled in the analyses. If applicable, describe which groupings were chosen and why	7, 8, 9
Statistical methods	12	(*a*) Describe all statistical methods, including those used to control for confounding	7, 8, 9
		(*b*) Describe any methods used to examine subgroups and interactions	7, 8, 9
		(*c*) Explain how missing data were addressed	7, 8, 9
		(*d*) *Cohort study*—If applicable, explain how loss to follow-up was addressed<br>*Case-control study*—If applicable, explain how matching of cases and controls was addressed	7, 8, 9

For peer review only

		Cross-sectional study—If applicable, describe analytical methods taking account of sampling strategy	
		(*e*) Describe any sensitivity analyses	7, 8, 9
**Results**			
Participants	13*	(a) Report numbers of individuals at each stage of study—eg numbers potentially eligible, examined for eligibility, confirmed eligible, included in the study, completing follow-up, and analysed	10, 11, 12
		(b) Give reasons for non-participation at each stage	10, 11, 12
		(c) Consider use of a flow diagram	10, 11, 12
Descriptive data	14*	(a) Give characteristics of study participants (eg demographic, clinical, social) and information on exposures and potential confounders	10, 11, 12
		(b) Indicate number of participants with missing data for each variable of interest	10, 11, 12
		(c) *Cohort study*—Summarise follow-up time (eg, average and total amount)	10, 11, 12
Outcome data	15*	*Cohort study*—Report numbers of outcome events or summary measures over time	10, 11, 12
		Case-control study—Report numbers in each exposure category, or summary measures of exposure	10, 11, 12
		Cross-sectional study—Report numbers of outcome events or summary measures	10, 11, 12
Main results	16	(*a*) Give unadjusted estimates and, if applicable, confounder-adjusted estimates and their precision (eg, 95% confidence interval). Make clear which confounders were adjusted for and why they were included	10, 11, 12
		(*b*) Report category boundaries when continuous variables were categorized	10, 11, 12
		(*c*) If relevant, consider translating estimates of relative risk into absolute risk for a meaningful time period	10, 11, 12
Other analyses	17	Report other analyses done—eg analyses of subgroups and interactions, and sensitivity analyses	10, 11, 12
**Discussion**			
Key results	18	Summarise key results with reference to study objectives	13
Limitations	19	Discuss limitations of the study, taking into account sources of potential bias or imprecision. Discuss both direction and magnitude of any potential bias	16
Interpretation	20	Give a cautious overall interpretation of results considering objectives, limitations, multiplicity of analyses, results from similar studies, and other relevant evidence	13-16
Generalisability	21	Discuss the generalisability (external validity) of the study results	13-16
**Other information**			
Funding	22	Give the source of funding and the role of the funders for the present study and, if applicable, for the original study on which the present article is based	23

*Give information separately for cases and controls in case-control studies and, if applicable, for exposed and unexposed groups in cohort and cross-sectional studies.

Note: An Explanation and Elaboration article discusses each checklist item and gives methodological background and published examples of transparent reporting. The STROBE checklist is best used in conjunction with this article (freely available on the Web sites of PLoS Medicine at http://www.plosmedicine.org/, Annals of Internal Medicine at http://www.annals.org/, and Epidemiology at http://www.epidem.com/). Information on the STROBE Initiative is available at www.strobe-statement.org.

For peer review only
